# Understanding the Positional Binding and Substrate Interaction of a Highly Thermostable GH10 Xylanase from *Thermotoga maritima* by Molecular Docking

**DOI:** 10.3390/biom8030064

**Published:** 2018-07-30

**Authors:** Jiangke Yang, Zhenggang Han

**Affiliations:** College of Biology and Pharmaceutical Engineering, Wuhan Polytechnic University, Wuhan 430023, China; jiangke.yang@gmail.com

**Keywords:** glycoside hydrolase family 10, xylanase, molecular docking, AutoDock, xylooligosaccharide

## Abstract

Glycoside hydrolase family 10 (GH10) xylanases are responsible for enzymatic cleavage of the internal glycosidic linkages of the xylan backbone, to generate xylooligosaccharides (XOS) and xyloses. The topologies of active-site cleft determine the substrate preferences and product profiles of xylanases. In this study, positional bindings and substrate interactions of TmxB, one of the most thermostable xylanases characterized from *Thermotoga maritima* to date, was investigated by docking simulations. XOS with backbone lengths of two to five (X2–X5) were docked into the active-site cleft of TmxB by AutoDock The modeled complex structures provided a series of snapshots of the interactions between XOS and TmxB. Changes in binding energy with the length of the XOS backbone indicated the existence of four effective subsites in TmxB. The interaction patterns at subsites −2 to +1 in TmxB were conserved among GH10 xylanases whereas those at distal aglycone subsite +2, consisting of the hydrogen bond network, was unique for TmxB. This work helps in obtaining an in-depth understanding of the substrate-binding property of TmxB and provides a basis for rational design of mutants with desired product profiles.

## 1. Introduction

*endo*-β-1,4-Xylanases (EC 3.2.1.8) perform enzymatic depolymerization of xylan backbone to generate xylooligosaccharides (XOS) with a low degree of polymerization (DP) (xylobiose, X2; xylotriose, X3; xylotetraose, X4; xylopentaose, X5; xylohexaose, X6) and xyloses [1]. The enzyme has considerable potential in many applications, such as biobleaching of pulps [2], deinking of waste paper [3], improving the digestibility of animal feed [4], bread making [5], converting of plant biomass into biofuels [6], and producing prebiotics [7]. 

According to the Carbohydrate-Active Enzyme (CAZy) database [8], xylanases mainly belong to glycoside hydrolase families 10 and 11 (GH10 and GH11, respectively) [9]. GH10 xylanases show broader substrate versatility and higher activity toward substituted forms of xylan and short XOS than GH11 xylanases [10]. GH10 xylanases (in the catalytic domain) have a typical (β/α)_8_-barrel fold and cleave glycosidic bonds via a double displacement mechanism [10,11]. In the C-terminal side of the β-strands, a cleft extending along the entire length of protein mediates sugar binding. The cleft is called an active-site cleft or substrate-binding cleft [12]. The catalytic acid/base and nucleophile residues are situated at the center of the active-site cleft [13]. Amino acids on the inner wall of the cleft compose the so-called subsites, which are responsible for binding of xylose residues [12]. The subsites are labeled as negative and positive numbers for the subsites accommodating the nonreducing (glycone region) and reducing (aglycone region) ends of the xylan polymer, respectively [14]. The catalytic residues are located between −1 and +1 subsites. Structural data combined with kinetic activity toward XOS generally indicated that approximately four to seven xylose binding sites exist in the active-site cleft of GH10 xylanases [12]. Highly conserved −2, −1, and +1 subsites play a crucial role in substrate binding [15,16]. In general, the subsites in the glycone and aglycone regions interact with xylose residues in distinct patterns. The glycone subsites bind to xylose residues through abundant hydrogen bonds, whereas the aglycone subsites anchor xylose residues mainly through hydrophobic stacking interactions [17,18]. 

Although the GH10 xylanases share a similar overall fold and catalytic mechanisms, the polymorphisms of subsites, in particular of the distal regions of active-site cleft, result in the different substrate binding preferences [18]. The substrate binding modes and product profiles depend on the topologies of the substrate-binding cleft. Many attempts have been made to determine the crystal structures of GH10 xylanases in complex with XOS to investigate the interactions between the xylopyranosyl ring and subsites. However, the complex structures showing an intact (uncleaved) XOS stretches over the −1 and +1 subsites are limited because the long-chain XOS (>3 xylose residues) were cleaved during crystal soaking or cocrystallization. In addition, inactive mutants of xylanases (glutamine, serine, or cysteine substitutions of one of the catalytic glutamates) should be applied for crystallization to avoid cleavage (Protein Data Bank (PDB) entries: 1E5N, 1UQY, 4L4P, 5GQE, 4PRW).

In this study, we showed that snapshots of GH10 xylanases and XOS interaction can be rapidly obtained by in silico study. TmxB, a GH10 xylanase produced by *Thermotoga maritima* MSB8, is one of the most thermostable GH10 xylanases characterized to date [19,20,21]. TmxB can efficiently hydrolyze xylan into short XOS, mainly X2 and xylose [20,21]. A three-dimensional structure (PDB entry: 1VBU) and a structure of TmxB in complex with X2 have been determined (PDB entry: 1VBR) [21]. In the complex structure, an X2 molecule was bound at subsites −2 to −1. Unusually, the −2 xylose residue exhibited a reverse conformation compared with that of many other crystal structures of GH10 xylanases in complex with X2 (PDB entries: 3MSD, 3NJ3, 5GQD) [21]. XOS with DP of two to five were docked into the substrate-binding cleft of the enzyme to understand the substrate-binding property of this xylanase with considerable application prospect. Our results indicated that four valid subsites −(−2 to +2) exist in the substrate-binding cleft of TmxB. Consistent with other GH10 xylanases, subsites −2 to +1 in TmxB were critical for xylopyranosyl ring interactions. The +2 subsite consisting of an arginine and a serine anchored xylopyranosyl ring through hydrogen bonds rather than hydrophobic stacking interactions, which are general among GH10 xylanases. Our study helps in obtaining an in-depth understanding of substrate recognition of this thermostable xylanase and provides a basis for the rational design of TmxB with new properties.

## 2. Results 

### 2.1. Amino Acid Sequence Comparison of TmxB and Other GH10 Xylanases

**Figure 1:**
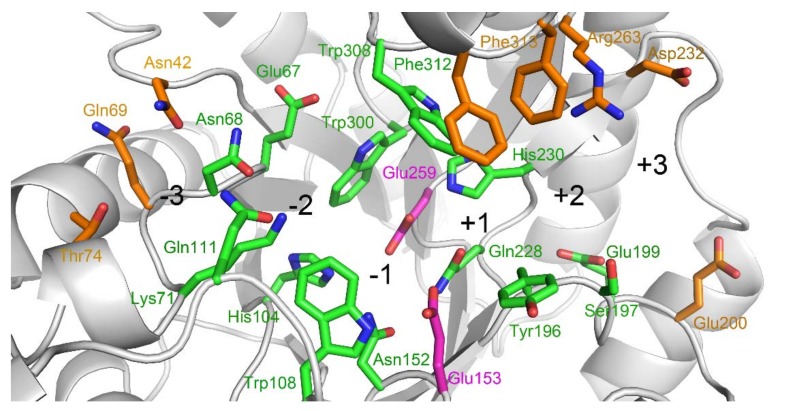
Substrate-binding cleft of xylanase from *Thermotoga maritima* (TmxB). The putative subsites are indicated by the large black numbers. The amino acids constituting each subsite are shown. The colors of the carbon atoms of the catalytic acid/base Glu153 and nucleophile Glu259 are shown in magenta. For the other amino acids, the colors of the carbon atoms are shown according to their degree of conservation, with green and brown for conserved and variable amino acids, respectively.

**Figure 2:**
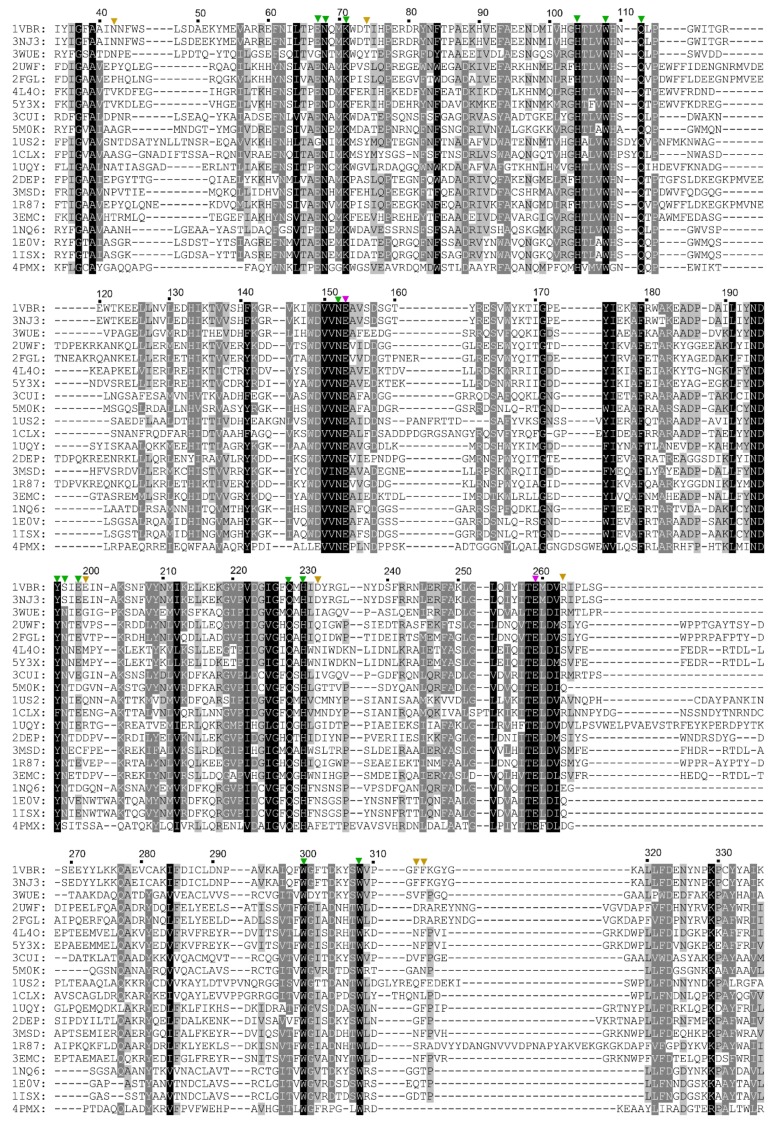
Multiple amino acid sequence alignments of the catalytic domains of TmxB and other structure-determined GH10 xylanases. Conserved amino acids are highlighted in black (strictly) and gray (semi-conservative). The amino acid sequences were obtained from the GenBank protein database [22]. The four characters before the sequences are the Protein Data Bank (PDB) entries of the corresponding protein structures of the xylanases. IVBU, TmxB (same amino acid sequence as the PDB entry 1VBR mentioned in the text); 3NJ3, xylanase 10B from *Thermotoga petrophila* RKU−1; 3WUE, XynAS9 from *Streptomyces* sp. 9; 2UWF, alkaline active xylanase from *Bacillus halodurans*; 2FGL, alkaline thermostable xylanase from *Bacillus* sp. NG-27 (BSX); 4L4O, xylanase from *Caldicellulosiruptor bescii* (*Cb*Xyn10B); 5Y3X, xylanase from *Caldicellulosiruptor owensensis* (*Co*XynA); 3CUI, xylanase from *Cellulomonas fimi* (*Cf*Xyn10A); 5M0K, xylanase from *Cellulomonas flavigena* DSM; 1US2, xylanase from *Cellvibrio japonicus* Ueda107 (*Cj*Xyn10C); 1CLX, xylanase from *C. japonicus* Ueda107 (*Cj*xyn10A); 1UQY, xylanase from *Cellvibrio mixtus* (*Cm*Xyn10B); 2DEP, xylanase from *Clostridium stercorarium*; 3MSD, intracellular xylanase from *Geobacillus stearothermophilus* (IXT6) (same amino acid sequence as the PDB entry 3MUA mentioned in the text); 1R87, extracellular xylanase from *G. stearothermophilus* (XT6) (same amino acids sequence for the PDB entry 4PUD mentioned in the text); 3EMC, xylanase from *Paenibacillus barcinonensis* (*Pb*Xyn10B); 1NQ6, xylanase from *Streptomyces halstedii*; 1E0V, xylanase from *Streptomyces lividans*; 1ISX, xylanase from *Streptomyces olivaceoviridis* E-86 (same amino acid sequence as the PDB entries 5GQD and 1V6V mentioned in the text); 4PMX, xylanase from *Xanthomonas citri*. The amino acid number of TmxB is labeled above the sequence. The acid/base catalyst and catalytic nucleophile are indicated by a magenta triangle. The conserved (conservative, and semiconservative) and variable amino acids are indicated by green and brown triangles, respectively. Gaps are denoted by dashes.

The crystal structure of TmxB has been determined (PDB entry: 1VBU) [21]. Basing on the structure, we could infer that the amino acids that pointed their side chains toward the active-site cleft may be involved in xylopyranosyl ring interaction in TmxB (Figure 1). Amino acid sequence and structure comparisons between TmxB and other representative GH10 xylanases revealed the different conservation levels of these amino acids (Figure 2 and Figure S1). The conserved amino acids were distributed throughout the −1 (His104, Trp108, Asn152, Glu153, Gln228, His230, Glu259, and Trp308) and −2 (Glu67, Asn68, Lys71, Gln111, and Trp300) subsites as well as part of +1 (Tyr196) and +2 (Ser197 and Glu199) subsites. Variable amino acids were mainly located at the putative subsites, −3 (Asn42, Gln69, and Thr74), +1 (Phe312), +2 (Arg263 and Phe313), and +3 (Glu200 and Asp232). Two consecutive aromatic amino acids (Phe312 and Phe313) and Arg263 in the aglycone region of active-site cleft were relatively unique for TmxB. These amino acids may play important roles in the substrate binding.

### 2.2. Docking Structure of X2-Bound TmxB

**Figure 3:**
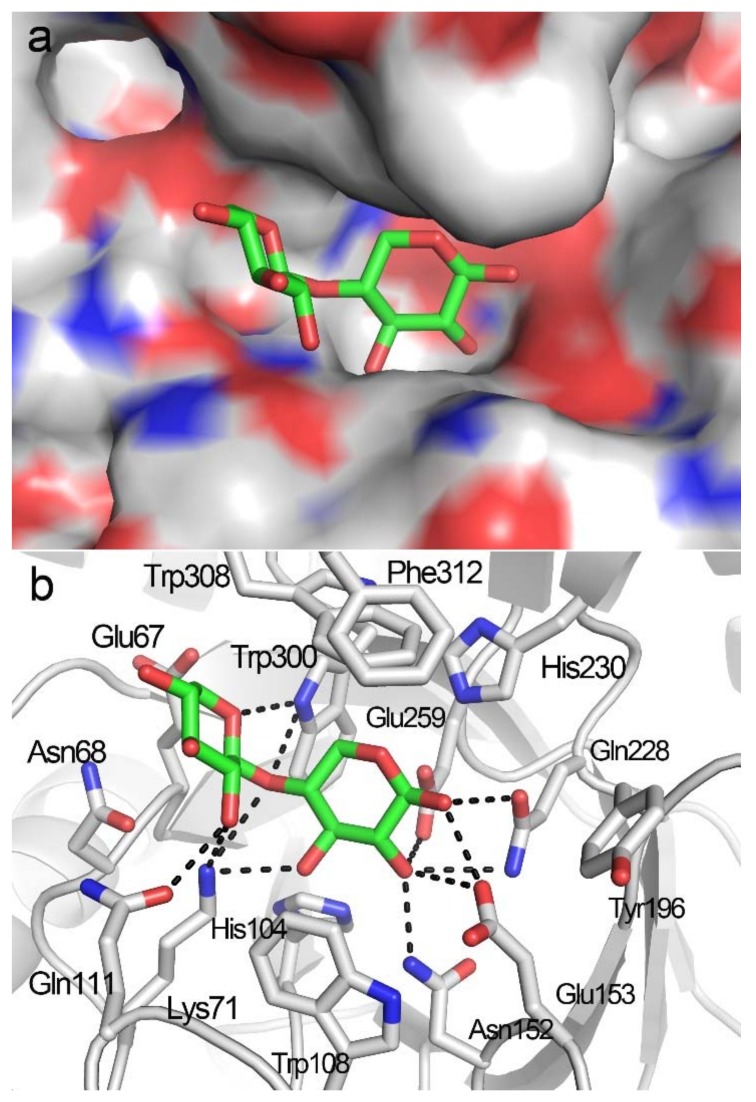
Docking structure of TmxB in complex with X2. (**a**) Binding position of X2 in the active-site cleft of TmxB in the docking structure. (**b**) Interaction details between TmxB and X2 molecule in the docking structure. The putative hydrogen bonds are indicated by black dashes. The carbon atoms of amino acids and X2 are shown in gray and green, respectively.

Although a bulky grid box, including the entire active-site cleft (subsites −3 to +3), served as the platform for X2 docking, the resulting docking poses indicated that the X2 molecule was only bound to the −2 to −1 subsites (Figure 3a). The lowest binding energy of the docking structures was −7.90 Kcal/mol. The −2 to −1 binding locations for X2 were detected in the crystal structures of TmxB-X2 itself (PDB entry: 1VBR) and complex structures of other GH10 xylanases in complex with X2 (PDB entries: 3MSD, 3NJ3, 5GQD), indicating a key role for subsites −2 to −1 in substrate recognition and binding [15,16]. Specifically, at the −1 subsite, amino acids Lys71, Asn152, Glu153, and Gln228 formed hydrogen bonds with xylose residue, and His104, Trp108, His230, Glu259, and Trp308 interacted with −1 xylose residue through hydrophobic interaction; at the −2 subsite, Lys71, Gln111, Trp300, and were involved in hydrogen bond interactions, and Glu67 and Asn68 interacted with xylose residues through hydrophobic interaction (Figure 3b and Figure S2). 

### 2.3. Docking Structure of X3-Bound TmxB

**Figure 4:**
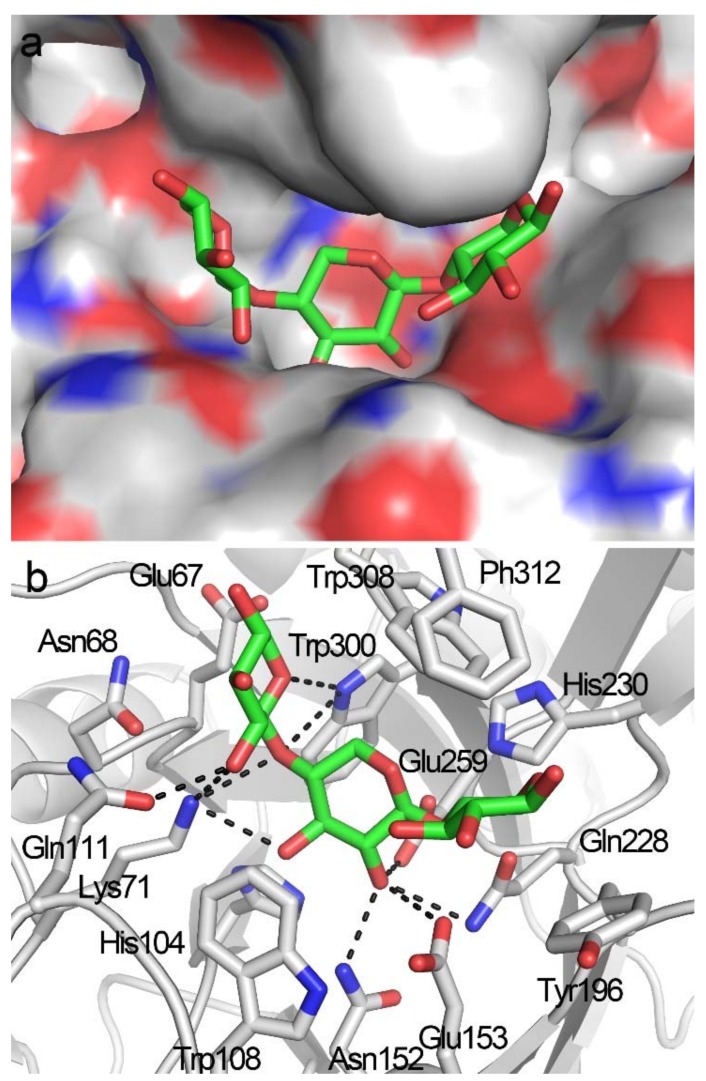
Docking structure of TmxB in complex with X3. (**a**) Docking conformations of X3 in the active-site cleft TmxB. (**b**) Interaction details between TmxB and X3 in the docking structure. The putative hydrogen bonds are indicated by black dashes. The carbon atoms of amino acids and X3 are shown in gray and green, respectively.

A −2 to +1 positional binding was observed when X3 was docked into the active-site cleft of TmxB (Figure 4a) and exhibited the lowest binding energy of −9.78 Kcal/mol. In the docking structure, xylose residues at subsites −2 and −1 exhibited conformations that were similar to those observed in the docking structure of TmxB-X2 (Figure 3b and Figure 4b, Figures S2 and S3). The xylose residue at the +1 subsite formed a hydrophobic stacking interaction with Tyr196 that was consistent with the interaction manner of the +1 subsite revealed by many crystal structures (PDB entries: 1R87, 1V6V, 3WUE) (Figure S1). Phe312 at the opposite site of Tyr196 interacted with +1 xylose residue through hydrophobic interaction (Figure S3). However, the aromatic ring of Phe312 was perpendicular to the xylopyranosyl ring.

### 2.4. Docking Structure of X4-Bound TmxB

**Figure 5:**
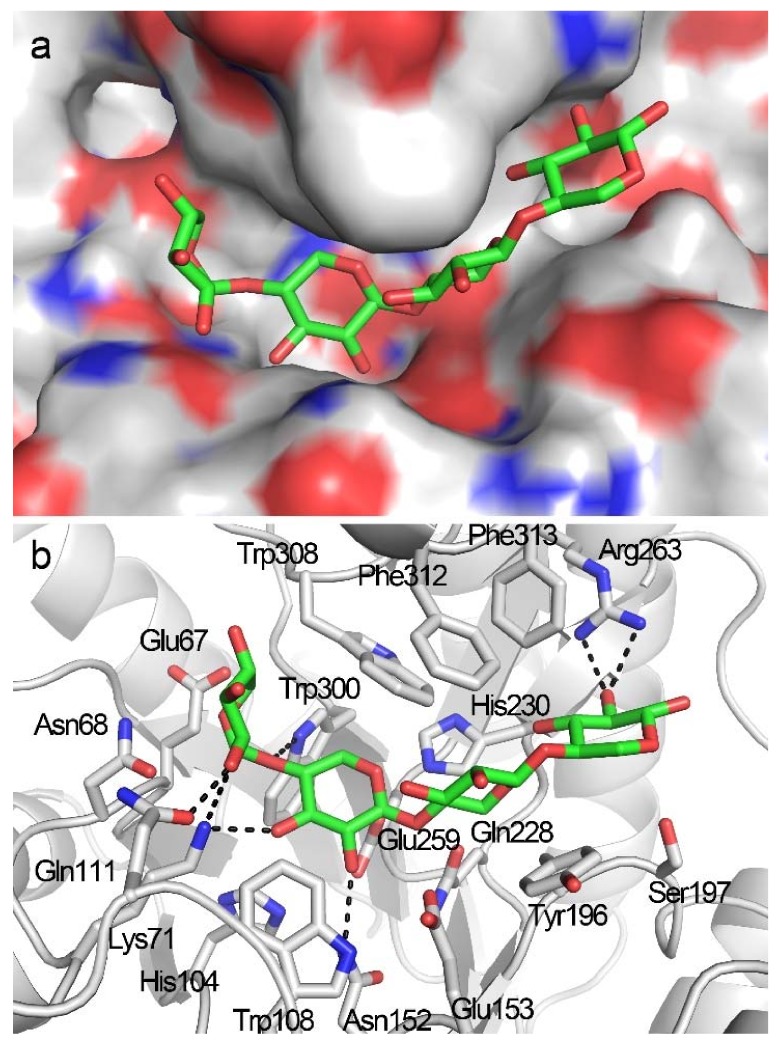
Docking structure of TmxB in complex with X4. (**a**) The overall binding location of X4 in the active-site cleft of TmxB. (**b**) Interaction details between TmxB and X4. The putative hydrogen bonds are indicated by black dashes.

The docking complex of TmxB-X4 with the lowest docking energy (−11.65 Kcal/mol) indicated that X4 was bound to the −2 to +2 subsites (Figure 5a). The conformation of xylose residues at the −2 to +1 subsites were largely consistent with that of the docking structure of TmxB-X3 (Figure 4b and Figure 5b). The +2 xylose residue in TmxB-X4 interacted with Arg263. The guanidine group in the side chain of Arg263 formed hydrogen bonds with OH-C2 of the +2 xylose residue (Figure 5b and Figure S4). The aromatic amino acid Phe313 at the +2 subsite did not form a hydrophobic stacking interaction with the xylopyranosyl ring (Figure 5b). 

### 2.5. Docking Structure of TmxB-X5 Complex

**Figure 6:**
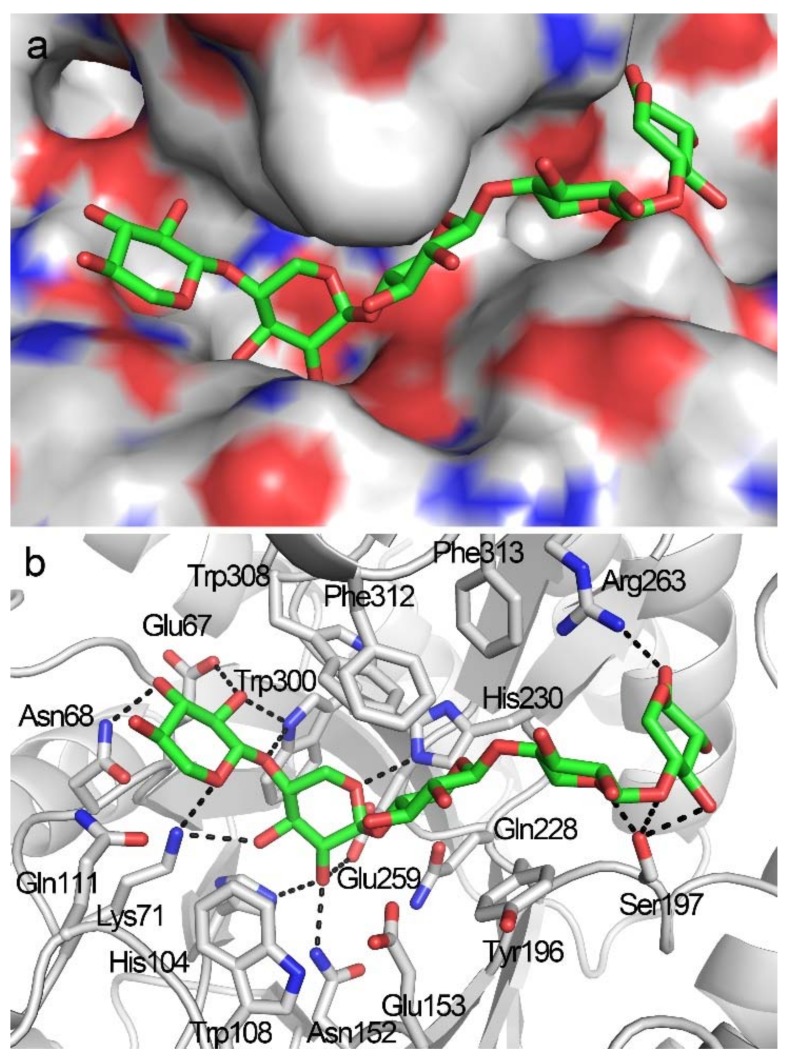
Docking structures of TmxB in complex with X5. (**a**) Binding position of X5 in the active-site cleft of TmxB. (**b**) Interaction details between TmxB and X5. The putative hydrogen bonds are indicated by black dashes. The carbon atoms from amino acids and X5 molecule are shown in gray and green, respectively.

Docking simulation indicated that the X5 molecule occupied the −2 to +3 subsites, which perfectly fitted with the shape of the active-site cleft in TmxB (Figure 6a). The docking complex exhibited the lowest docking energy of −11.48 Kcal/mol, comparable with that of the TmxB-X4 complex. Three xylose residues at the −2 to +1 subsites adopted a canonical conformation binding to the active-site cleft of GH10 xylanases (Figure 6b and Figure S1). The −2 xylose residue displayed a reverse conformation compared with that in the docking complex of TmxB-X2, TmxB-X3, and TmxB-X4 (Figure 3b, Figure 4b, Figure 5b and Figure 6b). The +2 xylose residue changed its orientation relative to that in the TmxB-X4 complex, resulting in dissociation from Arg263 and making polar contacts with Ser197 (Figure 5b and Figure 6b and Figure S5). The +3 xylose residue formed several hydrogen bonds with Arg263 and Ser197.

## 3. Discussion

The polymorphisms of subsites in the active-site cleft result in different substrate preferences and product profiles of individual GH10 xylanases [12]. Amino acid sequence comparison between TmxB and other GH10 xylanases revealed that subsites −2 to +1 in TmxB were conserved among the GH10 xylanases. The most significant variations were present in the relatively distal aglycone region of the active-site cleft of TmxB. Non-conserved amino acids in the region may be related to the substrate-binding property of TmxB. 

**Figure 7:**
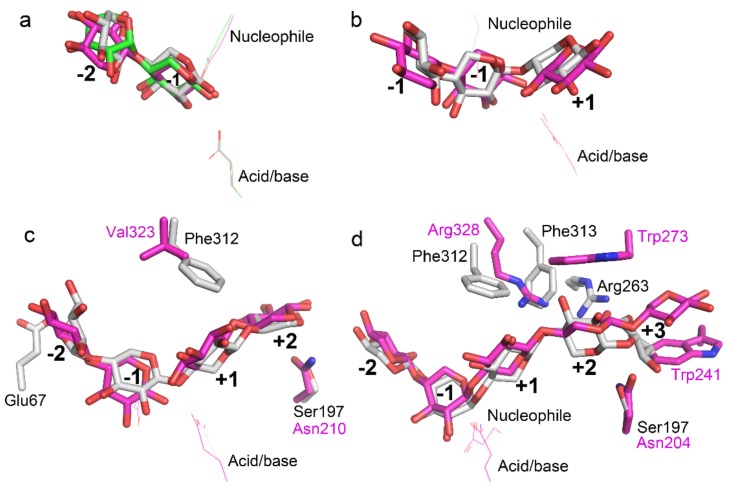
Superposition of TmxB xylooligosaccharide (XOS) (docking) and determined complex structures. (**a**) Superposition of the docking structure of TmxB-X2 (gray), crystal structure of TmxB-X2 (green, PDB entry:1VBR), and xylanase 10B from *T. petrophila* RKU-1 in complex with X2 (magenta, PDB entry: 3NJ3). (**b**) Superposition of docking structure of TmxB-X3 (gray) and crystal structure of intracellular xylanase from *G. stearothermophilus* (IXT6) in complex with X3 (magenta, PDB entry: 3MUA). (**c**) Superposition of the docking structure of TmxB-X4 (gray) and crystal structure of XynAS9 from *Streptomyces* sp. 9 in complex with X4 (magenta, PDB entry: 3WUE). (**d**) Superposition of the docking structure of TmxB-X5 (gray) and crystal structure of extracellular xylanase from *G. stearothermophilus* (XT6) (E159Q) in complex with X5 (magenta, PDB entry: 4PUD). The catalytic acid/base and nucleophile are shown in lines. The different amino acids between two compared xylanases are shown in sticks. The same color scheme was used to present the carbon atoms and label the amino acids.

In this study, XOS with backbone lengths of two to five was docked into the substrate-binding cleft of the enzyme to characterize substrate-binding in TmxB. Although a number of docking structures categorized into different conformational clusters were generated by AutoDock (Molecular Graphics Laboratory, La Jolla, CA, USA) [23], only the lowest energy docking poses of each XOS were applied to structural analysis because they represented the most possible binding modes of individual XOS. The docking simulations indicated that XOS can be properly docked into the active-site cleft. The shortest XOS, that is, X2, was bound to the nonreducing subsites −2 to −1, in agreement with the observation that no xylosidase activity was detected for TmxB [20]. The −2 to −1 binding modes were consistent with those observed in the crystal structures of X2 complexes (PDB entries: IVBR and 3NJ3) (Figure 7a). The binding position of X3 was located at the −2 to −1 subsites in the docking structure of TmxB-X3. Such a binding mode was observed in the crystal structures of IXT6 (intracellular xylanase from *Geobacillus stearothermophilus*) in complex with X3 (cleaved, with X2 and xylose located at −2 to −1 and +1 subsites, respectively) (PDB entries 3MUA and 3MSG) (Figure 7b). The positional bindings of X2 and X3 were limited to the regions by the nearly strictly conserved amino acid compositions at the −2 to +1 subsites (Figure 1 and Figure 2). X4 was bound to the −2 to +2 subsites in TmxB, as indicated by docking simulation. The same X4 binding mode was observed in the crystal structure of XynAS9 in complex with X4 (cleaved, with two X2 located at the −2 to −1 and +1 to +2 subsites) (PDB entry: 3WUE) (Figure 7c). The subsite topology of XynAS9 from −2 to +2 subsites was similar to that of TmxB, except for a glutamate/glycine substitution at the −2 subsite, and phenylalanine/valine and serine/asparagine substitutions at aglycone subsites (Figure 7c). A binding mode of −2 to +3 for X5 was observed in the docking structure of the TmxB-X5 complex. The same positional binding was observed in the crystal structure of XT6 (extracellular xylanase from *G. stearothermophilus*) (E159Q) in complex with X5 (PDB entry: 4PUD), although the amino acid composition at the aglycone subsites were significantly variable between TmxB and XT6 (Figure 7d). Arg263 and Ser197 in TmxB and aromatic amino acid in XT6 underwent the main interaction with xylose at the +3 subsite, which resulted in the preference for the −2 to +3 binding mode rather than the −3 to +2 binding modes in these two structures. Docking simulation of a long XOS (X6) showed that X6 bound to TmxB in a −3 to +3 mode (data not shown). However, for the lowest energy docking pose, the XOS chain adopted a reversed direction (the reducing and non-reducing ends of X6 interacted with the glycone and aglycone subsites, respectively). Moreover, in most docking poses, the non-reducing end of X6 bent, which led to an unreasonable interaction between xylose residues and amino acids outside of the substrate-binding cleft. This result can be ascribed to an absence of, or a weak −3 subsite in TmxB.

Structural analysis of the docking complexes revealed additional details of the interaction between XOS and TmxB. In all of the docking structures, the conformations of −1 xylose residues were highly uniform, and no obvious differences were observed for the −1 subsite interactions between TmxB and other GH10 xylanases (Figure 7 and Figure S1). The −2 xylose residue exhibited a nearly reversed conformation (with its OH-C2 and OH-C3 groups pointing to the outside of the active-site cleft) in the docking structures of TmxB-X2, TmxB-X3, and TmxB-X4 compared with the general conformation of −2 xylose residue observed in the crystal structures (Figure 7 and Figure S1). As a result of this inversion, the −2 xylose residue lost the hydrogen bond interaction with Asn68. The inverse xylose was interesting because a similar conformation for −2 xylose residue was observed in the crystal structure of TmxB in complex with X2 (Figure 7a) (PDB entry: 1VBR) [21]. However, the electron density map of the −2 xylose residue in this crystal structure is ambiguous. In the docking structure of TmxB-X5, the −2 xylose residue adopted its canonical conformation and interactions. The +1 xylose residue in all of the docking structures (TmxB-X3, TmxB-X4, and TmxB-X5) formed a hydrophobic stacking interaction with a tyrosine (Tyr196), consistent with that of the crystal structures of GH10 xylanases (Figure 7 and Figure S1). 

The docking structures indicated that the xylose residues at the more distal aglycone subsite +2 and +3 interacted with the polar amino acids (Arg263 and Ser197) through the hydrogen bond network. Such an interaction pattern was different from the predominantly hydrophobic stacking interactions between GH10 xylanases and substrates at aglycone subsites [12]. For example, the interactions at the distal aglycone subsites in the well-investigated XT6 were dominated by hydrophobic stacking interactions, as indicated by the crystal structure of XT6-X5 (PDB entry: 4PUD) (Figure 7d). The +2 and +3 xylose residues adopted different conformations because of the different amino acid compositions at the +2 to +3 subsites between TmxB and XT6. In the XT6-X5 complex, the positions and orientations of +2 and +3 xylose residues were fixed by forming hydrophobic stacking interactions with Trp273 and Trp241, respectively (Figure 7d). 

**Figure 8:**
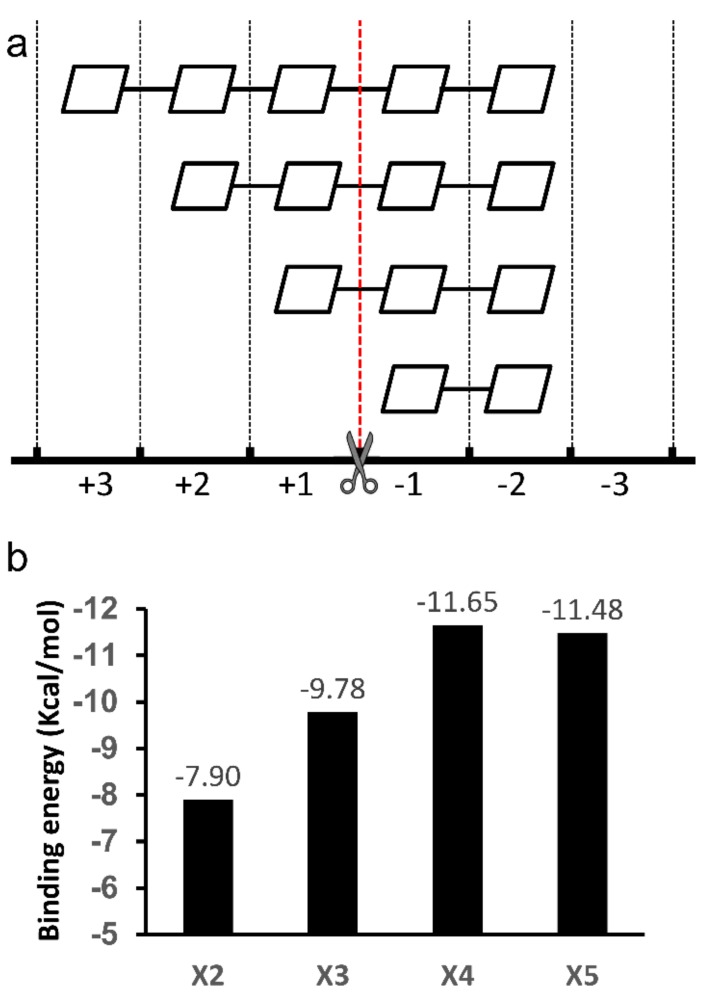
Summary of the binding modes of xylooligosaccharide (XOS) in TmxB. (**a**) Proposed positional binding of XOS to the active-site cleft in TmxB on the basis of the docking structures. The cleavage position is indicated by a scissor. (**b**) The binding energy changes with the backbone length of XOS.

The overall XOS positional binding and corresponding cleavage pattern in TmxB are shown in Figure 8a. The −2 to +1 cleavage of X3 by TmxB had been demonstrated by thin-layer chromatography [21]. The binding energy of each docking complex indicated the affinity between the individual XOS and TmxB. When the XOS chain stretched one xylose residue, the binding energy (the lowest for each XOS) increased by approximately −2 Kcal/mol (Figure 8b). The increasing tendency terminated in X5, indicating the existence of four effective xylopyranosyl ring binding sites in TmxB. According to the binding modes observed by docking simulations (Figure 8a), these subsites were −2 to +2. Normally, such a deduction was made on the basis of thermodynamic experiments. For instance, isothermal titration calorimetry showed that each additional xylose residue led to an increase in binding energy and the increase trend continued to X6, indicating the existence of six subsites in XT6 [17] and *Cb*Xyn10C from *Caldicellulosiruptor bescii* [24].

## 4. Materials and Methods

### 4.1. Amino Acid Sequence and Structure Comparison of TmxB and Other GH10 Xylanases

The amino acid sequences and the three-dimensional structures of TmxB and other representatives of GH10 xylanases were downloaded from the GenBank protein database [22] and PDB [25,26], respectively. Twenty GH10 xylanases were selected for multiple sequence and structure alignments. The multiple sequence alignment was conducted using Clustal Omega [27] and structure comparison was carried out using PyMol (Schrödinger, New York, NY, USA).

### 4.2. Receptor Molecule Preparation for Docking

The crystal structure of TmxB (PDB entry: 1VBU) was used as the receptor for the docking simulations. Waters in the structure were removed, and the structure was minimized using UCSF (University of California, San Francisco) Chimera [28]. The NE1 and NE2 atoms of histidine, NE, NH1, and NH2 atoms of arginine, NZ atom of lysine, NE1 atom of tryptophan, and NE2 atom of glutamine were set as protonation, whereas the OE1 atom of glutamine, and the OE1 and OE2 of atoms glutamate were treated as deprotonation. The residues in 1VBU were renumbered according to the corresponding full-length protein sequence of TmxB deposited in UniProtKB [29] (accession number: Q9WXS5).

### 4.3. Preparation of the Three-Dimensional Structure of XOS

Three-dimensional structures of XOS with backbone lengths of 2 to 5 were used as ligands for docking. Their structures were obtained from deposited structure complexes of GH10 xylanase in PDB. Specifically, X5, X4, X3, and X2 were from PDB entries 1E5N, 1US2, 4L4P, and 3NJ3, respectively. 

### 4.4. Docking Siulations and Analysis

Enzyme-ligand docking simulations were conducted using the AutoDock Tools 1.5.6 platform (ADT) [22]. A grid box of size 42 × 30 × 70 points was applied to all XOS. The box encompassed the entire active-site cleft with +1 and −1 subsites at the center. The receptor molecule was treated as rigid, and the ligands as flexible. The maximum number of energy evaluations was set to 25,000,000 (10 times the default). The other parameters were set as default. The resulting docking structures were ranked according to their binding energy scored by the function of ADT (based on the United Atom version of the AMBER force field) [30]. The lowest energy conformation was used for positional binding analysis. The criterion for hydrogen bond judgment was that the maximum distance between donor and acceptor atoms should be less than 3.4 Å. Each docking simulation was performed three times and the same results were obtained. The complex structures were visualized and analyzed using PyMol (Schrödinger) and LigPlot [31]. 

## 5. Conclusions

The amino acid sequence and structure alignments showed that the aglycone subsites in TmxB were nonconserved. Molecular docking revealed the roles of these subsite amino acids in positional binding and xylose residue interactions. Theoretical complexes obtained by docking of XOS (X2–X5) into the active-site cleft of TmxB showed that the −2 to +2 subsites in TmxB played a critical role in substrate binding. The +2 subsites anchored the xylose residue through a hydrogen bond network rather than the general hydrophobic stacking interaction at the aglycone subsites in GH10 xylanases. On the basis of substrate interactions observed in this in silico study, the consequences of mutations at the subsites could be predicted, which helps in alerting the character of TmxB though rational design. In addition, our results indicated the effectiveness of docking simulation in investigating the interaction between glycoside hydrolase and short oligosaccharides.

## Supplementary Materials

The following are available online at http://www.mdpi.com/2218-273X/8/3/64/s1, Figure S1: Structure alignment of representative GH10 xylanases. Figure S2: Ligand interaction diagram of the docking complex of TmxB-X2 drawn by LigPlot. Figure S3: Ligand interaction diagram of the docking complex of TmxB-X3 drawn by LigPlot. Figure S4: Ligand interaction diagram of the docking complex of TmxB-X4 drawn by LigPlot. Figure S5: Ligand interaction diagram of the docking complex of TmxB-X5 drawn by LigPlot.
