# Supplementary material for: Understanding the Positional Binding and Substrate Interaction of a Highly Thermostable GH10 Xylanase from Thermotoga maritima by Molecular Docking
Source: Biomolecules. 2018 Jul 30;8(3):64. doi: 10.3390/biom8030064 (PMC6163442; doi:10.3390/biom8030064)
Supplement: Supplementary file 1 [file biomolecules-08-00064-s001.pdf]

Supplementary data

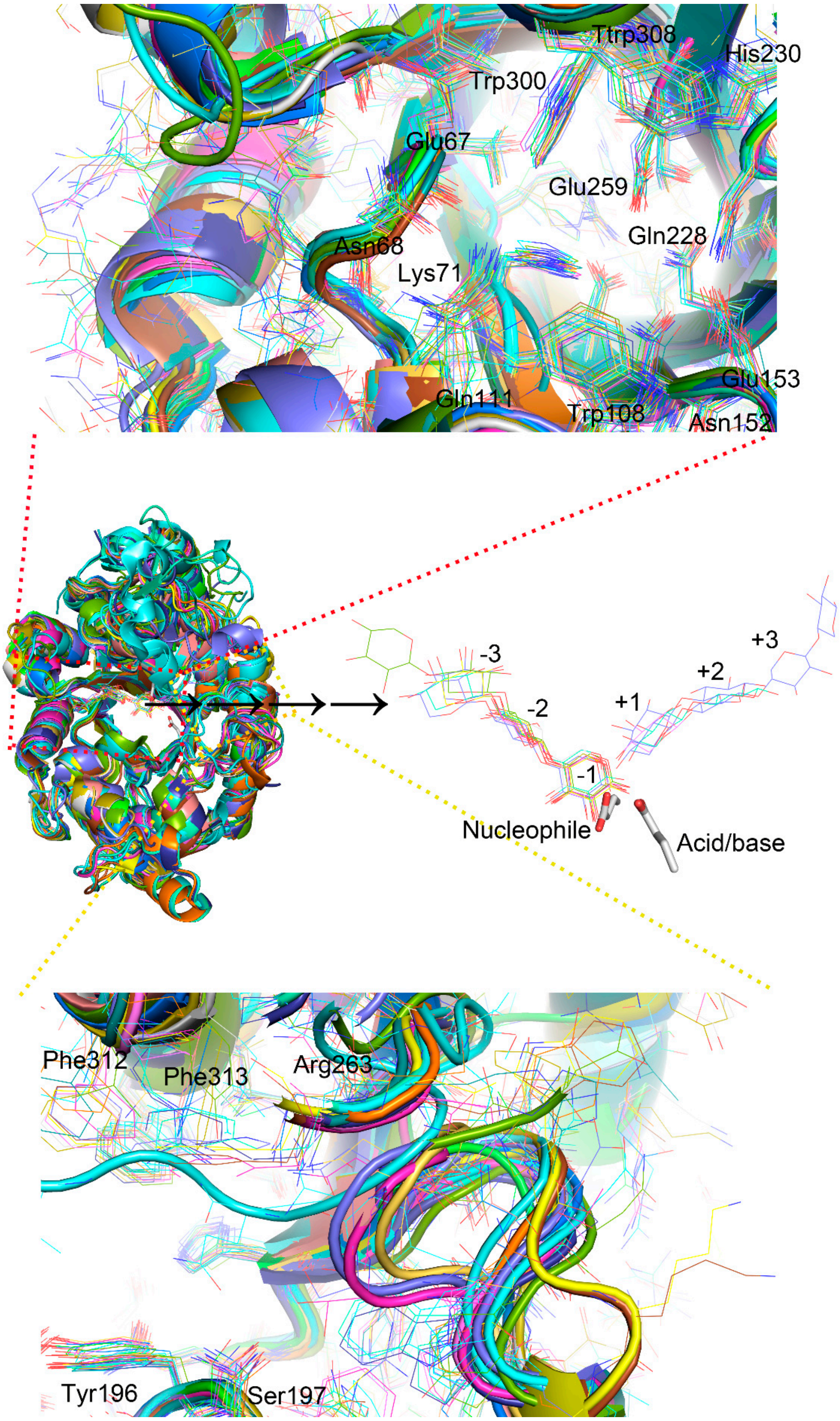

Figure S1 Structure alignment of representative glycoside hydrolase family 10 xylanases. The coordinates used for structural alignment correspond to the amino acid sequences in Figure 2 of main text. The coordinates include following Protein Data Bank (PDB) entries. IVBR, xylanase from *Thermotoga maritime* (TmxB); 3NJ3, xylanase 10B from *Thermotoga petrophila* RKU-1; 3WUE, XynAS9 from *Streptomyces* sp. 9; 2UWF, alkaline active xylanase from *Bacillus halodurans*; 2FGL, alkaline thermostable xylanase from *Bacillus* sp. NG-27 (BSX); 4L4O, xylanase from *Caldicellulosiruptor bescii* (*Cb*Xyn10B); 5Y3X, xylanase from *Caldicellulosiruptor owensensis* (*Co*XynA); 3CUI, xylanase from *Cellulomonas fimi* (*Cf*Xyn10A) (same amino acids sequence for the PDB entry 3CUJ mentioned in the text); 5M0K, xylanase from *Cellulomonas flavigena* DSM 20109; 1US2, xylanase from *Cellvibrio japonicus* Ueda107 (*Cj*Xyn10C); 1CLX, xylanase from *Cellvibrio japonicus* Ueda107 (*Cj*xyn10A); 1UQY, xylanase from *Cellvibrio mixtus* (*Cm*Xyn10B); 2DEP, xylanase from *Clostridium stercorarium*; 3MSD, intracellular xylanase from *Geobacillus stearothermophilus* (same amino acids sequence for the PDB entry 3MUA mentioned in the text); 1R87, extracellular xylanase from *Geobacillus stearothermophilus* (XT6) (same amino acids sequence for the PDB entries 4PUD, 4PUE, and 4PRW mentioned in the text); 3EMC, xylanase from *Paenibacillus barcinonensis* (*Pb*Xyn10B); 1NQ6, xylanase from *Streptomyces halstedii*; 1E0V, xylanase from *Streptomyces lividans*; 1ISX, xylanase from *Streptomyces olivaceoviridis* E-86 (same amino acids sequence for the PDB entries 5GQD and 1V6V mentioned in the text); 4PMX, xylanase from *Xanthomonas citri*. Close-up views of glycone subsites (up), aglycone subsites (bottom), and ligands (XOS) in the substrate-binding cleft (right). The conserved amino acids in the substrate-binding cleft are labeled (TmxB numbers). The poses of XOS in the subsites illustrate the general catalytic mechanism of GH10 xylanases. One glutamate (acid/base) acting as acid catalyst provides proton for glyosidic oxygen and another glutamate (nucleophile) locating down below anomeric carbon of the -1 xylose residue attacks the anomeric carbon, and forms a glycosyl enzyme intermediate. The pose of -1 xylose can be used as a judging rule to exclude the irrational docking poses.

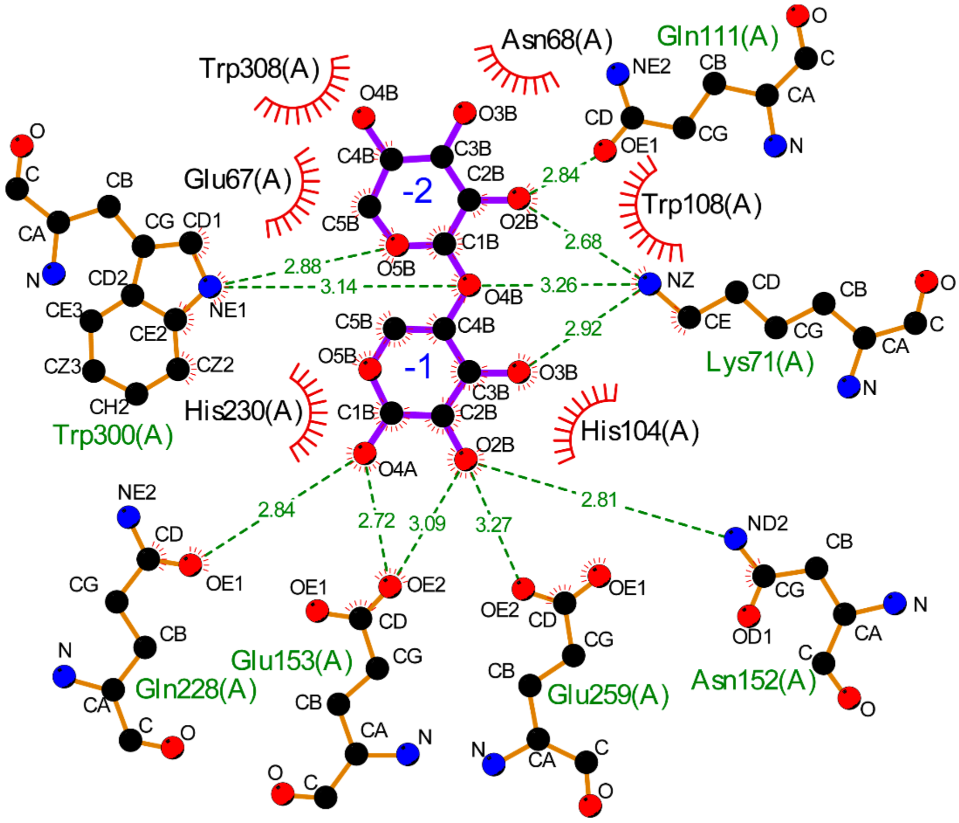

Figure S2 The ligand interaction diagram of xylanase from *Thermotoga maritima* (TmxB) in complex with xylobiose (X2) drawn by LigPlot. The amino acids involved in hydrogen bond and hydrophobic/Vander Waals interactions are labeled in green and black, respectively.

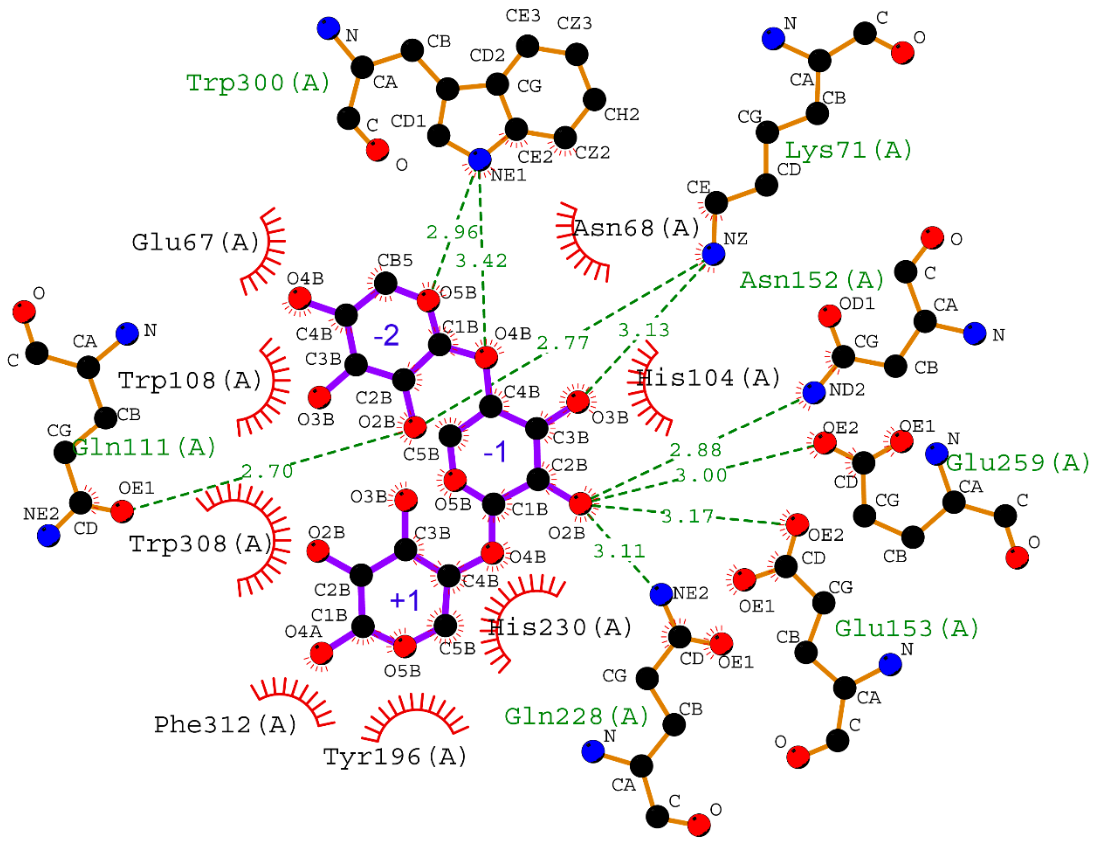

Figure S3 The ligand interaction diagram of xylanase from *Thermotoga maritima* (TmxB) in complex with xylotriose (X3) drawn by LigPlot. The amino acids involved in hydrogen bond and hydrophobic/Vander Waals interactions are labeled in green and black, respectively.

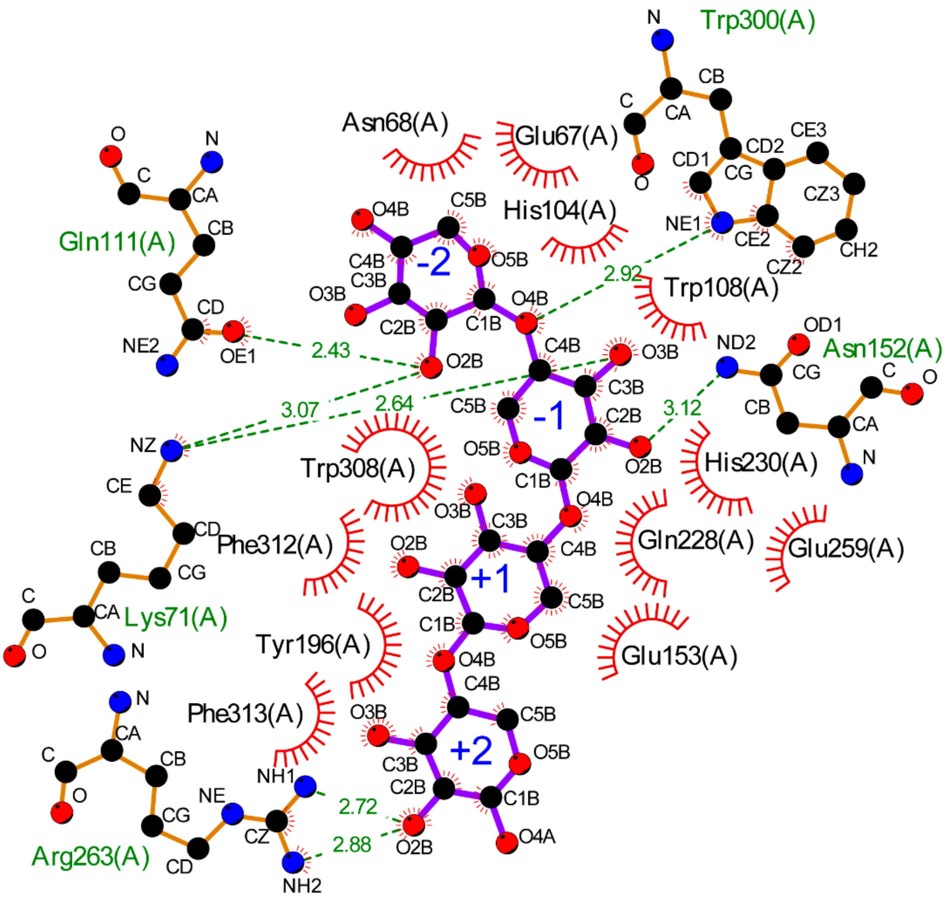

Figure S4 The ligand interaction diagram of xylanase from *Thermotoga maritima* (TmxB) in complex with xylotetraose (X4) drawn by LigPlot. The amino acids involved in hydrogen bond and hydrophobic/Vander Waals interactions are labeled in green and black, respectively.

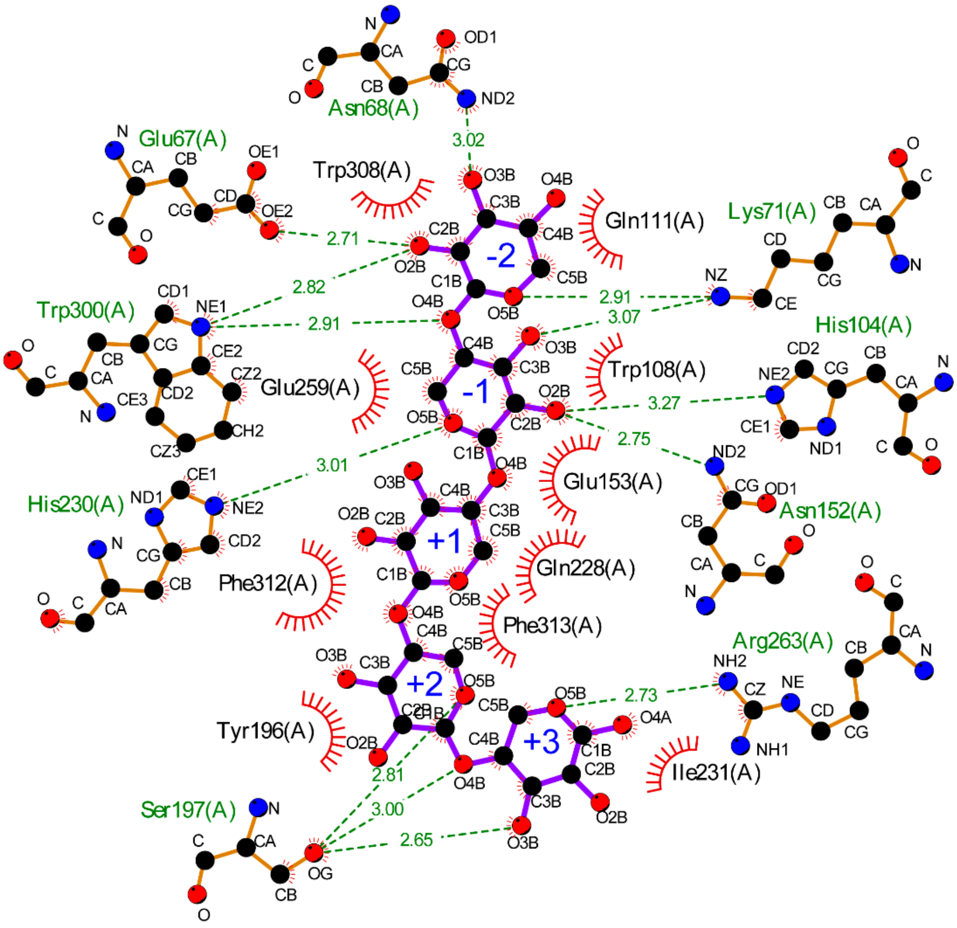

Figure S5 The ligand interaction diagram of xylanase from *Thermotoga maritima* (TmxB) in complex with xylopentaose (X5) drawn by LigPlot. The amino acids involved in hydrogen bond and hydrophobic/Vander Waals interactions are labeled in green and black, respectively.
